# ‘There’s Just Something Really Peaceful About It’: a Qualitative Exploration of Mothers with Young Children and Engagement in Group-Based Physical Activity Programs

**DOI:** 10.1007/s12529-022-10062-0

**Published:** 2022-02-17

**Authors:** Louisa R. Peralta, Zali Yager, Ivanka Prichard

**Affiliations:** 1 School of Education and Social Work, Faculty of Education and Social Sciences, The University of Sydney, Sydney, NSW Australia; 2 Institute for Health and Sport, Victoria University, Melbourne, 3011 Australia; 3 Health & Exercise Sciences, College of Nursing & Health Sciences, Flinders University, Adelaide, South Australia Australia

**Keywords:** Physical activity, Postpartum women, Group-based physical activity programs, Maintenance, Facilitator, Maternal health

## Abstract

**Background:**

Many mothers with young children often do not achieve recommendations of at least 150-min moderate-to-vigorous physical activity (MVPA) each week. Previous qualitative work has generally focused on getting inactive mothers with young children to be active, so the characteristics of women who are active during early postpartum period are not well understood. This research set out to capture the characteristics of mothers with young children who engage in MVPA and how these women manage barriers and harness enablers to sustain in engagement in physical activity (PA) over an extended period.

**Method:**

Thirty-two participants ranging in age from 27 to 42 years (35.2 ± 4.8), with age of their youngest child ranging from 6 weeks old to 5 years, participated in semi-structured interviews.

**Results:**

Inductive thematic analysis revealed three overarching themes and fourteen sub-themes relating to the characteristics of active mothers with young children and the engagement and maintenance factors that recruit and sustain these women in group-based physical activity programs. Specifically, mothers with young children relish a welcoming and supportive environment that accommodates babies and young children, is affordable and convenient, focuses on building strength and functionality, and is non-judgmental.

**Conclusion:**

These findings advance knowledge by providing considerations and recommendations that support intervention and program designers to be able to develop group-based physical activity programs for mothers with young children.

## Introduction

The health benefits of physical activity are well established and include a lower risk of cardiovascular disease, hypertension, diabetes, and breast and colon cancer and positive effects on mental health [1, 2]. Despite the known benefits, many adults do not meet the recommended international health guidelines of 150 min of moderate-to-vigorous physical activity (MVPA) accumulated over the course of a week, including 2 days of muscle-strengthening activities [3]. Thirty to forty percent of Australian women do not meet the Australian PA guidelines [3], which decreases again with mothers who have young children [4, 5]. Evidence shows that there are several individual, social, and environmental factors that act as barriers and/or facilitators for mothers with young children when aiming to resume or begin MVPA [6–8]. Individual and social factors include income, number of children, working around baby feeding and nap times/routines, fatigue, being able to make time for themselves, a lack of social support, and negative family attitudes and beliefs about parenting [7, 9]. In addition, research has also highlighted body image, shame, and body (dis)functionality as individual barriers to PA after childbirth [10]. Other social and environmental barriers include access to public transport, access to recreational facilities with adequate child care, PA programs with appropriate progressions for a safe return to MVPA, neighbourhood safety concerns, and lack of access to an informative health system [7, 9]. Interventions targeting individual and environmental factors to support postpartum (i.e. most often defined up to 2 years after birth; see [11]) MVPA have demonstrated mixed success, with a meta-analysis finding that postpartum PA interventions had a short-term moderate-sized effect on women’s MVPA frequency but no effect on overall volume [11]. Clearly, mothers with young children represent an important demographic group for targeted promotion of MVPA.

Women’s postpartum MVPA trajectories vary; many women decrease MVPA, but some women maintain previous MVPA, and a small percentage increase their MVPA in the early postpartum period [12]. Recently, Liva et al. [13] qualitative study of 30 healthy mothers of infants aged 2 to 12 months explored mothers with young children’s MVPA decision-making to identify characteristics and reasonings of active mothers with young children. Findings reported diverse rationales for MVPA engagement, showing complexity in the ways mothers with young children construct their MVPA choices. Interestingly, participants who regarded MVPA as central to their wellbeing and identity were more likely to engage in MVPA. These findings fit with a small amount of previous qualitative research with mothers with young children; that is, those with higher self-efficacy tend to be better able to overcome individual and environmental constraints and to engage in regular MVPA [9]. Saligheh et al. [9] also highlight that social expectations and taking cues from past MVPA experiences encourage women to make positive MVPA decisions in the early postpartum period.

Research to date has helped to identify intention and self-efficacy as important constructs to target in interventions, but this literature still contains noteworthy limitations. First, most research has focused on inactive or overweight/obese mothers with young children or with comorbid conditions (e.g. diabetes and/or postnatal depression) [11, 14]. This makes previous findings difficult to generalise to mothers with young children who commence PA programs. Second, current research tends to focus on lifestyle programs that target several health behaviours, rather than single-focused PA programs. Hence, the research often lacks details of the types of PA programs that healthy mothers with young children are most likely to successfully engage in [11, 13, 14]. Therefore, more research is needed to determine the characteristics of mothers with young children who are active (i.e. self-report that they are engaged in at least 150 min of MVPA/week), the types and factors of PA programs that they engage in, and the various points during the postpartum period that these women make decisions to manage barriers and harness enablers to sustain their engagement in PA over an extended period.

One important social factor for supporting women with young children is social support [7, 9]. Group-based interventions may be an effective way to increase MVPA among high-risk populations and those most in need of health behaviour intervention [15]. A recent systematic review and narrative synthesis found only six studies that reported on the effect of group-based PA interventions and mothers with young children’s MVPA or other behavioural and anthropometric outcomes [16]. Findings report that these studies were moderately successful in changing or increasing mothers with young children self-reported MVPA levels and psychological wellbeing in the first 2 years of their children’s lives. However, the characteristics and factors of group-based PA programs that enhanced mothers with young children’s MVPA and psychological wellbeing were not made clear.

With these limitations in mind, the purpose of the present study was to use qualitative methods to explore and develop an understanding of (1) the characteristics of active mothers with young children with children aged 0–5 years (defined as the postpartum period in this study); (2) factors that encourage mothers with young children to join group-based PA programs; and (3) factors that sustain mothers with young children’s participation in group-based PA programs. In doing so, the study aims to inform PA policies and support intervention and program designers to be able to develop group-based PA programs for mothers with young children in the future.

## Materials and Methods

### Design and Setting

The research was carried out between September and December 2020 in the surrounding regions of Sydney (including Newcastle and Wollongong) and Wagga Wagga, Australia. Semi-structured interviews were conducted to explore women’s characteristics, views and experiences relating to group-based PA programs during the postpartum period. Individual interviews were conducted using video-conferencing software (Zoom®) or face-to-face in a convenient location for both parties and utilised COVID-safe procedures which were in place at the time (e.g. physical distancing). Zoom® interviews were suitable in this population, due to COVID-19 restrictions, as well as being less burdensome for participants who may have found it hard to find the time to attend a face-to-face interview. All interviews were conducted by the first author (LP). Interview recordings were taken using a password-protected mobile phone recording device and saved as an audio (MP3) file. Written informed consent and verbal assent were obtained before each interview. MP3 files were transcribed verbatim by a transcription company and were checked for accuracy by the interviewer and by participants. Ethical approval was gained from the University of Sydney research ethics committee (Ref 2020/540).

### Sample Characteristics

Women were eligible to take part if they were aged 18 years and over, engaged in a group-based PA program, lived in Australia, spoke fluent English, and had at least one biological child less than or 5 years old before the study recruitment date.

Thirty-two participants ranged in age from 27 to 42 years (35.2 ± 4.8), with age of the youngest child ranging from 6 weeks old to 5 years. Participants were well-educated (71.9% had at least a Bachelor’s degree) and were predominantly of Caucasian origin (84.4%). Two participants identified as Southern European, one as Aboriginal and Southern European, one as Asian, and one as Middle Eastern. Based on suburb postcode, 53.1% of participants lived in a suburb with a mean decile rank that was below the median on the Australian Bureau of Statistics’ Index of Relative Socioeconomic Disadvantage (https://www.abs.gov.au/websitedbs/censushome.nsf/home/seifahelpansuis).

Most women interviewed had two children (65.6%), followed by three children (18.8%). Five women (15.6%) had one child. Most women were in part-time employment in professional jobs (87.5%), with one woman returning to full-time employment (3%). In addition to employment, two were studying and most were married or living with a partner (90.6%). Two participants had previously competed in elite level sport (6%). Table 1 provides an outline of participant details.

**Table 1:** Characteristics of the participant sample

**Participant no.**	**Age**	**No. children**	**Age of children (in years unless otherwise stated)**	**Single (S)/married (M, which includes living with partner)**	**Work status (full-ime [FT]/part-time [PT]/no work [NW])**	**Type of group-based PA program (high-intensity interval training [HIIT]/sport [S]/running [R])**
**1.**	40	2	5, 3	S	PT	HIIT
**2.**	32	2	3, 1	M	PT	HIIT
**3.**	36	3	7, 4, 6 weeks	M	PT	HIIT
**4.**	29	1	9 months	M	PT	HIIT
**5.**	31	2	3, 1	M	PT	HIIT
**6.**	42	3	9, 7, 3	M	PT	HIIT
**7.**	41	2	5, 3	M	PT	HIIT
**8.**	40	3	7, 5 (stillborn), 3	M	PT	HIIT
**9.**	29	2	5, 10 months old	M	NW	HIIT
**10.**	33	2	3, 1	M	NW	HIIT
**11.**	38	2	6, 4	M	PT	HIIT
**12.**	27	1	6 months	M	PT	HIIT
**13.**	30	1	7 months	M	PT	HIIT
**14.**	38	2	5, 3	M	PT	HIIT
**15.**	36	2	4, 3	M	PT	HIIT
**16.**	37	2	6, 3	M	PT	R
**17.**	28	2	3, 10 weeks	M	PT	HIIT
**18.**	40	2	11, 3	M	PT	HIIT
**19.**	35	1	2	S	PT	HIIT
**20.**	40	1	4	M	PT	HIIT
**21.**	37	2	6, 4	S	PT	S
**22.**	34	2	4, 2	M	PT	HIIT
**23.**	37	2	4, 2	M	PT	S
**24.**	40	2	5, 3	M	PT	HIIT
**25.**	28	3	9, 6, 1	M	PT	HIIT
**26.**	39	3	10, 4, 1	M	PT	HIIT
**27.**	38	2	4, 1	M	PT	HIIT
**28.**	35	2	7, 5	M	PT	S
**29.**	41	2	5, 3	M	FT	HIIT
**30.**	27	2	3, 1	M	PT	HIIT
**31.**	39	2	6, 5	M	PT	HIIT
**32.**	28	3	5, 4, 1	M	NW	HIIT

### Procedure

A purposive sampling and variation sampling methods [17] were used for recruitment, which was carried out through publicly available contact details (e.g. social media) of group-based PA programs that targeted mothers with young children. Participants were recruited from a range of group-based programs (e.g. group-based high-intensity interval training [HIIT] classes, organised sport, organised running groups) to ensure that a broad range of different experiences and perceptions was identified. The sample size was dependent on theoretical saturation [18], with interviews conducted until saturation was achieved, leading to a final sample of 32 mothers with young children (a large sample size for a thematic analysis; 19), with an average interview time of 33.45 min (ranged from 18.41 to 47.29 min). The interview guide was developed based on previous literature and expertise within the research team. The interview guide was piloted on an active mother with one child in the 0–5-year age group. Questions were consistent across all interviews (see Table 2 for the iterative interview guide); however, flexibility was encouraged to enhance the authenticity of each woman’s response (i.e. questions were brought forward if participants introduced these ideas in their earlier responses).

**Table 2:** Interview guide questions

**Question No.**	**Questions plus prompts**
**1.**	To what extent has physical activity (PA) been important to you during your life?
**2.**	Tell me about your PA levels before children? Were you happy with the amount and type of exercise you were doing?
**3.**	Tell me about your PA levels during pregnancy? Were you happy with the amount and type of exercise you were doing?
**4.**	What type of birth experience did you have? How has this affected your return to PA?
**5.**	Tell me about the postpartum PA program that you are currently attending regularly? Why did you start attending?
**6.**	Tell me about your PA levels in the first couple of months after the birth of your baby? Prompt: Can you tell me how you felt when participating in PA?
**7.**	Tell me how has the postpartum PA program enabled your current PA levels? Prompt example: activities/exercises that are programmed
**8.**	Can you describe the internal enablers that have helped with maintaining your engagement with the postpartum PA program? Prompt example: feelings of belonging to a group
**9.**	Have you experienced any challenges that have minimised your engagement with the postpartum PA program during this time? Prompt example: How have you overcome these challenges?
**10.**	Tell me the extent of how your engagement in the postpartum PA program has influenced other parts of your life (e.g. health and wellbeing; parenting and child rearing)?
**11.**	How has your engagement with the postpartum PA program influenced your children? Do they attend sessions? How many? What do they do during this time?
**12.**	If there was one piece of advice you would give to those people designing and implementing postpartum PA programs for Mums, what would it be?
**13.**	What would you ‘call’ or ‘name’ this PA now that you are a mum?

### Analysis

The interviews were transcribed verbatim, and any identifying information was removed from the transcripts, and participants were assigned pseudonyms. The transcriptions and data were shared and managed via Dropbox. The analysis involved six phases: (1) familiarisation with the data; (2) generating initial codes; (3) searching for themes; (4) reviewing themes; (5) defining and naming themes; and (6) producing the report [19]. Initial coding was undertaken by the first author and ZY on a small sample of transcripts (*N* = 3). Code lists were compared between the two authors (85% agreement), and any discrepancies between codes were resolved by consensus resulting in an initial coding framework. The first author then coded all interview transcripts. This stage involved prolonged engagement with the data, including reading all transcripts, listening to audio files, editing transcripts for accuracy, and then re-reading for immersive comprehension. Deductive codes were created for key concepts from existing theoretical and research literature (i.e. characteristics of active mothers with young children; experiences of mothers with young children’s experiences of group-based PA programs; and features of group-based PA programs that sustain mothers with young children’s PA levels), and inductive codes were developed in response to patterns identified in the data. Multi-coding of the same text was allowed, where important codes overlapped on the same dialogue. The codes were then grouped according to similarity of topic and used to form potential themes. All authors individually developed theme names that captured all codes included within the theme, with theme names discussed when discrepancies occurred.

## Results

Themes presented in this paper focus on describing the characteristics of mothers with young children, their experiences of group-based PA programs, and the features of group-based PA programs that maintain their engagement. The themes are presented as considerations for the design of future PA programs for mothers with young children: Theme 1: Women do not need to have a strong PA history; Theme 2: Engagement factors that recruit mothers with young children into group-based PA programs; and Theme 3: Maintenance factors that sustain mothers with young children’s participation. Table 3 outlines the three themes, sub-themes, and prevalent codes derived for each sub-theme.

**Table 3:** Mothers with young children and group-based physical activity programs: themes, sub-themes, and prevalent codes

**Themes/sub-themes**	**Prevalent codes**
**Theme 1. Women do not need to have a strong PA history**	
* 1.1 PA in childhood and adolescence*	- PA and sport focus throughout life - Sport in adolescence
* 1.2 Previous participant in group-based PA programs*	- Not a gym participant previously - Gym participant previously
* 1.3 Birth and health check-ups*	- Easing back into PA postpartum - 6-week check as a milestone
**Theme 2. Engagement factors that recruit mothers with young children into group-based PA programs**	
* 2.1 Connection*	- Friends in the group - Word of mouth
* 2.2 Family support*	- Social support/family support - Role model for family and community
* 2.3 Convenience/location/proximity*	- Location and proximity to home - Previous experiences with PA/sport
* 2.4 Cost and affordability*	- No contract fees/not locked in - Memberships
* 2.5 Welcoming ethos*	- Initial hesitance, first group-based program - Building confidence
**Theme 3. Maintenance factors that sustain mothers with young children’s participation in group-based PA programs**	
* 3.1 Child friendly*	- Kid friendly - Being able to balance baby and PA
* 3.2 Body positive*	- Expectations of postpartum body - Competence and feelings of confidence
* 3.3 Role modelling for children*	- Intention/hope - Kids copying/joining in on sessions
* 3.4 Social and psychological benefits*	- Benefits, mental health - Social-forming/maintaining friendships

### Theme 1: Women Do Not Need to Have a Strong PA History

Participants outlined their engagement in PA before and during pregnancy to determine the role PA played in their lives. Three sub-themes captured the extent of PA variation across the lifespan: (1.1) PA in childhood and adolescence; (1.2) previous group-based PA program experience; and (1.3) birth and health check-ups. Across sub-themes, it was apparent that women’s PA history varied, with some participants *(n* = 11; 34%) shaped by past PA behaviours and experiences. Each participant was subject to unique personal experiences relating to birth(s), and this allowed a slow, progressive return to PA (at least 12 weeks post-birth). Interestingly, women in this study were not heavily invested in group-based PA programs prior to bearing children.

#### Sub-theme 1.1: PA in Childhood and Adolescence

All except two of the participants were engaged in sport and physical activities during childhood and adolescence. Some of the participants mentioned that engagement in sport and physical activities decreased once they finished school (e.g. ‘but when I finished school I stopped…I was not interested’ (Jackie)), started tertiary study (e.g. ‘As a child I participated in dancing. And I did that, probably from eight years old till I was eighteen and then I stopped when I started studying’ (Diane)), or when balancing work commitments (‘And then once I entered into the workforce, it probably cut back a little bit’ (Natalie)). The frequency of participation in sport and physical activities during childhood and adolescence differed among the participants. Some of the participants (*n* = 12; 38%) represented (played at a higher level than recreational sport) in at least one sport, which meant that they engaged in training and games most days of the week. Others were recreational participants who participated a couple of times per week. The types of sport and physical activities varied. Some had been engaged in individual sports such as dance, gymnastics, triathlon, tennis, and swimming; others were engaged in team or group sports such as netball, volleyball, and soccer and a few had been engaged in a combination of both individual and group sports and PA. Vivienne shows her strong intentions for PA throughout adolescence and into early adulthood and how this decreased before having children:

Quite high intensity. I would go running. Go to a kickboxing class. Sometimes gym, although I’m more of an outdoor person. Play netball or touch football. That was more when I was younger though, as opposed to probably just prior to having kids.

#### Sub-theme 1.2: Previous Group-Based PA Program Experience

Many participants (*n* = 20; 63%) seemed to have previous experiences with gym-based activities (e.g. group-based classes, boot camps) during early adulthood after ceasing or reducing sport participation and balancing study/work commitments. Rachel encapsulates this transition from study to work and how she was able to maintain PA through group-based sessions at her workplace gym:

So I’ve always been very active and then working at the uni, I’ve always been a member of the uni gym, so I can go into a group activity class at lunchtime…so pretty active when you look at it.

A few of the participants acknowledged that they needed to be active in group situations, even before bearing children, for motivational purposes. Without the group context, these participants believed that they were less likely to be consistently and regularly active: ‘And I think that tends to be affected by my motivation levels. I tend to be more motivated in group settings’ (Laura). However, not all were previous group-based PA program participants before having children, and some did not like the gym environment. For example, Louise showed her previous distaste for gyms and group-based PA sessions as she disliked the environment: ‘One, I hated gyms. And two, I could never commit to anything’.

#### Sub-theme 1.3: Birth and Health Check-ups

The participants, despite their varied birthing experiences, attended and completed the 6-week health check-ups conducted by their choice of medical personnel (e.g. doctor, obstetrician, gynaecologist). After this check-up, many of the participants started to engage in some type of PA, namely, walking. If the check-up showed that there were still pregnancy or birthing injuries, a few of the women in the study continued with physiotherapy and medical support and were very cautious when returning to PA, particularly in relation to MVPA:

Yeah. I took really good care of it and I probably ... I was more cautious. So [name of women’s health physiotherapist] would say, ‘I think you can run now’. And I’d be like, ‘No, another month’. So yeah, I didn’t run for 18 months after he was born. (Jessie)

There were a small number (*n* = 3) of physiotherapists in the participant sample. These participants stressed the importance of and the need to extend healthcare after birth and past the usual 12-week period, especially before resuming or starting high-intensity group-based PA programs:

I also saw a women’s health physio, before I started in the gym, even though I’m a physio myself. I went to her to get checked over so she could check my level of pelvic activation, and she was happy, she ran through a few tests to make sure that she was happy for me to go back and start in the gym again and I did that at 12 weeks. (Jemma)

### Theme 2: Engagement Factors that Recruit Mothers with Young Children into Group-Based PA Programs

Participants highlighted the strategies, people, costs, and environment that encouraged them to commence or resume group-based PA programs after the birth of their child(ren). These are captured in five sub-themes: (2.1) connection; (2.2) family support; (2.3) convenience/location/proximity; (2.4) cost and affordability; and (2.5) welcoming ethos. Across sub-themes, it was apparent that participants who were starting a group-based PA program utilised personal and professional social networks to connect with this new venture and perhaps without this connection would have missed out on this opportunity. It was also interesting that physical, social, and financial barriers relating to starting and sustaining participation in a group-based PA program were overcome, mainly through a welcoming environment, and were not considered an ongoing barrier.

#### Sub-theme 2.1: Connection

Most of the participants (*n* = 27; 84%) noted that it was a significant other in their life that informed them of the benefits of participating in a group-based PA program after having child(ren). The significant other was likely to be a family member, friend, or associate (e.g. friend of a friend, neighbour) who knew of, was going to try, or was already a participant of a group-based PA program. For most of these women, it was an important introduction to the program at a time when juggling young child(ren) made it near impossible for them to find the time and the motivation to try a new program by themselves:

And [my neighbour] said, you know what, I’ve started going to [group-based PA program name]...There’s a couple of other girls that I’ve just met. It’s a nice way for you to meet people…it was an introduction to a peer group of people and friendships when I was quite lonely and isolated because of little kids. (Vivienne)

The other women resumed participation in group-based PA programs that they were engaged in before having child(ren). Hence, their PA choice was shaped by past PA behaviours, although now needing to be modified due to early motherhood.

#### Sub-theme 2.2: Family Support

Many of the women (*n* = 20; 63%) highlighted those supportive others were also an important key for participation in a group-based PA program. Specifically, these women noted that baby development and routines placed significant demands on their time and limited their participation in regular group-based PA program sessions, but it was usually a supportive family member that put strategies in place to help overcome such obstacles. These included grandmothers babysitting at home when the baby/toddler napped, ‘So my mum actually used to come and let [baby’s name] sleep…and I’d still get to go to the [group-based PA program name]’ (Melanie), and partners staying home or starting work late/leaving work early to accommodate the women’s wishes to get to a session: ‘Because [husband’s name] is such a good support for me, where he will hold back before leaving for work, so that I can get a workout into the day’ (Lottie).

Family support was particularly important for women that resumed participation in organised, group-based sport that did not cater for babies and young children (e.g. in-built babysitting). These women were extremely appreciative of supportive family members who would attend, watch, and supervise their baby/child(ren), with the acknowledgement that without family support, their participation would be minimised:

My parents still enjoy watching me play organized sports, so they were there and there’d be a plethora of kids, including mine around and Mum and Dad would babysit. Otherwise it would be really difficult to participate in [group-based sport]. (Belinda)

Due to the demands women felt they were placing on family to support their participation in group-based PA programs and the sustainability of this, most women favoured places and spaces that incorporated babysitting and child-minding. This was emphatically emphasised in many (*n* = 27; 84%) of the participants’ responses:

…but the thing that really drew me in was that there were people there with their strollers. I was like, ‘Yes, I can go and take my baby along with me’. (Sharon)

#### Sub-theme 2.3: Convenience/Location/Proximity

Half of the women’s narratives (*n* = 17; 53%) revealed that the location of the group-based PA program was important, noting that their chosen group-based PA program was close to home or in a location where they preferred to be active (e.g. beach). These women mostly expressed a desire to be exercising outside, with their child/ren close by, in which they could easily walk to or commute in a car for a short period of time:

I just saw that as a good opportunity for me to be outdoors, at the beach and doing exercise with someone minding the baby. So, it ticked a lot of boxes. (Lucy)

I started to look for…like just a mum’s group, like exercise outdoors and I found [name of group-based PA program]. I’s only like a seven minutes drive from home. (Renee)

All women in this study stated that variety was a major factor in joining the group-based PA program, as it allowed for them to work around babies, child(ren), and other professional and personal commitments. They also enjoyed the convenience of having many choices of sessions (e.g. bootcamp, running, strength, stretch) and different timing of sessions per day and week or different levels and grades for organised sport:

I think that’s the variety of times that they offer too. So, you can go at six o’clock in the morning or you can go at six o’clock at night. There’s often the bit in the morning, a bit in the afternoon, or you can just go in, there’s a lot of sessions. So you can generally find the time. (Natalie)

Some of the participants (*n* = 4; 13%) preferred to be active early in the morning, without child(ren) and before the members of their household woke: ‘And this women’s running club just goes at 5:00 in the morning…I didn’t have to organize anybody or ask for help with looking after the kids’ (Claire). Having programs that had scheduled times early in the morning allowed for this to occur and should be considered in future group-based PA programs.

Another consideration highlighted by the mothers with young children was the length of the sessions and the need for it to be shorter to ensure that those who brought babies and child(ren) along would be able to do so without interrupting eating and napping schedules. However, these women also clearly communicated that the intensity would need be appropriate for shorter sessions to improve their fitness and strength, as it was paramount that these sessions rebuilt their functional capacity:

My favourites are the spin and high-intensity classes. They’re the 45-minute sessions, but I feel like I get a much bigger workout in those 45 minutes than going to a 60-minute bootcamp, which is important to me. (Lottie)

#### Sub-theme 2.4: Cost and Affordability

Many of the women (*n* = 30; 94%) spoke about the financial costs of participating in group-based PA programs, and that this was an important consideration when joining as most were on leave or working in a reduced capacity (e.g. returning to work part-time or casually). Some of the women explained the need for program designers and trainers to consider no contract, pay-as-you-go options as these allowed mothers with young children the flexibility to not worry about missing a session or week due to baby and child(ren) sickness or other personal and professional commitments:

And just the ease of not having that locked in contract, because that’s hard for a parent, especially a new mum to turn off, my baby didn’t sleep or is sick, but I’ve locked myself in and I have to go, because I have spent 50 bucks a week to go. While this way, I turn up and I apologize (if I have missed a session). (Rachel)

Alternatively, others stated that the contractual costs of a program were suitable by reinforcing that there were many attributes to the program that were included in the membership. These attributes included attending as many sessions as they chose each week, free babysitting/child-minding, and sharing the membership with their partner:

Yeah. I mean, it’s quite affordable considering the amount of things you can get out of that $40 per week. I can attend as many sessions as I like, or as little as I like. When my husband joined, they gave him a temporary discount, $70 for the both of us. (Jackie)

#### Sub-theme 2.5: Welcoming Ethos

Despite the participants’ ability to sustain their participation in a group-based PA program, their initial foray was mixed. Most started slowly after giving birth or resuming PA participation, but some of the other reasons for hesitance was due to uncertainty about the social environment, the physical capabilities needed to participate, and balancing baby and child(ren) routines with PA sessions. For example, for those who had only limited experiences in the gym or in a group-based situation prior to having child(ren), they attended once or twice a week until they had developed belief and confidence in their abilities and established social connections within the group and with the trainers:

Oh, it would’ve been once a week. Yeah, that was about all I felt comfortable doing until I figured my place in it all and until I felt comfortable doing the activities in the sessions. (Louise)

The number one external factor that helped women overcome their initial hesitancy and lack of confidence was the welcoming ethos that was prevalent in all the group-based PA program environments. The women spoke wholeheartedly about the supportive environment created by the trainers, the other participants, and the shared journey. They also downplayed the importance of the physical space, further enhancing the influence of a positive emotional and social environment for recruiting mothers with young children: ‘It doesn’t matter about equipment or space or anything like that. It’s all about the emotional and social connections with the trainers and other participants’ (Jemma). An important component of creating a welcoming ethos and a positive emotional and social environment for mothers with young children was the absence of peer comparison and judgement:

Well, I think that’s what most women want is you want someone who’s going to take the time to get to know you and what you can do and where your body’s at and not go and compare you to others. It’s somewhere that’s a safe environment that you can feel comfortable in going in six weeks postpartum or a year after you’ve had a baby. (Nicola)

### Theme 3: Maintenance Factors that Sustain Mothers with Young Children’s Participation in Group-Based PA Programs

The third theme was identified as participants explained their motivation for continuing to engage in group-based PA programs. Four sub-themes captured the personal and environmental factors that supported them: (3.1) child friendly; (3.2) body positive; (3.3) role modelling for children; and (3.4) social and psychological benefits. Although these sub-themes are different to the ones that helped women join group-based PA programs in the first place, it should be acknowledged that family support, convenience, location, affordability, and a welcoming ethos were still influential in sustaining participants’ participation. Across sub-themes, it was apparent that the women’s motivation to continually engage in the group-based PA sessions was usually tied to several internal and environmental motivational factors.

#### Sub-theme 3.1: Child Friendly

The main reason for the women in this study to be able to maintain their participation in a group-based PA program was that the environment allowed for babies/child(ren) to be in the same vicinity as the mother. Most of the women chose or had to bring their child(ren) to the PA space, so they appreciated that the program and trainers were accommodating of child(ren) and had constructed a space, physically and emotionally, that allowed mothers and child(ren) to have direct access to each other during the session in a safe manner:

And then it’s just knowing that I can take my kids. That is everything to me because I have to work part-time. It’s always been a thing for us financially that I’ve had to do. So, I don’t want to put them back in care…So, being able to take them with me and still have them play and have a good time and have access to different toys and me. (Vivienne)

The women in this study who brought their child(ren) to the PA space understood the PA sessions may be interrupted. However, there was also an understanding between trainers and mothers that this was reasonable, and that, if possible, the trainers would modify the session or help and support the mother with her child(ren). The environment was fluid and dynamic, which made the mothers in this study feel comfortable but also still benefit from being physically active:

I remember there was about a month straight where I’d bring [child’s name] and he just cried the whole time and the women in here are just so magical. They saw that I needed this exercise. I was struggling and they would stop what they were doing and go and take [child’s name] for a walk. I still remember, there was about three sessions where [name of trainer] just stroked [child’s name] head for 45 minutes straight. (Kristy)

#### Sub-theme 3.2: Body Positive

Motivation to continually engage in the group-based PA sessions was also attributed to an environment which promoted what the women’s body could do, rather than what their bodies looked like. Some of the women noted that this was a positive change from previous PA experiences before child(ren): ‘It always used to be about appearance for me, but now it’s more about functionality…And the [name of group-based PA program] does this’ (Kim).

Several women in this study shared specific examples of how the environment and the trainers were able to maintain the focus on the functionality of the body. The women stressed that the absence of mirrors, specific and individualised feedback on improvement in completing exercises or building strength, and modifications or progressions for focus be continually placed on body functionality and capability:

And [name of trainer] drives that self-satisfaction with her praise and feedback. And she always says to me, ‘Oh well, Anna, you know when you started, you couldn’t even squat two centimetres, and now you can almost squat this far’. So she always gives you feedback and praise, which I think is a big thing that you need. (Anna)

Despite the focus on the functionality of the body, some of the women still grappled with their postpartum body, often comparing it with the pre-baby body and highlighting the differences. However, when the women did this, there was also a positive frame in terms of being able to acknowledge its differences, as well as their postpartum body’s new capabilities. This highlights that the body image–exercise relationship varied during early motherhood for these participants but tended to be influenced by participation in a group-based PA program:

I guess just physically I’m doing things I never did pre-baby because I never went to the gym and stuff like that beforehand…And now I just feel really strong. My body shape has definitely changed and I feel strong... I definitely feel like I’m the fittest I’ve ever been and that’s post-baby and I never thought I would be like that. (Jessie)

#### Sub-theme 3.3: Role Modelling for Children

Most mothers with young children (*n* = 28; 88%) recognised that a motivating factor for sustaining their participation was having their child(ren) participating when possible. The reasons for this was the belief that the child(ren), especially those who were 2 years or older, were learning from being not only observers, but also active participants:

My oldest was, ‘Look at me, I can carry this one’. …And I think I can see connections with them mimicking what I’m doing. If they see my yoga mat lying around at home, next thing it’s rolled out on the floor and they’re doing push-ups and trying to count. (Lucy)

The role modelling theme was reinforced with the participants’ narratives highlighting an element of hope that their child(ren) would maintain these PA behaviours over an extended period or the child(ren) would recognise that participation was important for their mothers’ mental health and wellbeing. As P4 mentions: ‘Hopefully she understands that I’m doing this to be not only physically active, but for my own mental health and wellbeing as well’ (Laura). Interestingly, a few of the mothers with young children (*n* = 6; 19%) believed that they were also teaching the value of self-care to their child(ren) and emphasising that mothers also need to complete tasks and enjoy activities too:

…and the fact that the kids then put value on self-care. ‘This is mummy’s time to do mummy’s thing. You’ve got time and I will support you on that. Sometimes, its mummy’s turn is to do this’. … That skill is valuable too. (Belinda)

#### Sub-theme 3.4: Social and Psychological Benefits

The group-based PA program was used as a tool for enhancing physical, psychological, and social functioning during the early stages of motherhood. Rebuilding strength, fitness, and functional capacity, reinforcing positive thoughts and capabilities of the body, psychological stress relief, and social enjoyment were discussed as benefits and motivators of participating regularly. In fact, a few of the participants acknowledged that the social health benefits outweighed the physical benefits. This is made clear in Giselle’s statement ‘It’s almost secondary to me, the physical stuff. Yeah, it’s like that’s just a bonus of it all’.

In addition to the social benefits, many of the women claimed several mental health and wellbeing benefits. These included being ‘a happier mum and a happier wife’ (Lottie), ‘I feel like I’m ready for the day’ (Claire), ‘it’s my stress outlet’ (Kristy), and ‘it’s filling my cup first which then helps with my whole family’ (Natasha). For those who were living with a mental health condition, the group-based PA program was a mechanism for alleviating the symptoms or minimising or ceasing the intake of medication:

So, for me, I had to make exercise a priority because I didn’t want to be on medication. I’m healthy. I’m active…With my first child, I was stressed about being a mum. I was sleep-deprived and lots of different things. But then when I had my daughter, the anxiety never came back as exercise was a priority. (Cindy)

Some of the mothers with young children also used PA to gain relief from the requirements and expectations of being a mother. Some of the women explicitly spoke about this, as seen in Lily’s comment: ‘I think the biggest thing for me is, that keeps me going back is just the fact that it just gives me a little bit of independence, it gives me, the, me time, I get to be me instead of just mum. And that’s probably the biggest thing for me that keeps me going back’. However, it seems that most of the women used the group-based PA program as a resource rather than an escape or refuge. It was a time to share parenting situations, ask for help or ideas, and reflect on strategies used and shed emotions about parenting in a safe and supportive environment. This buoyed mothers with young children’s social and mental wellbeing as well:

Or, wow, I felt like such a bad mum because I yelled. And everyone’s like we have all done that. And then just being able to normalize those feelings. (Vivienne)

## Discussion

The aim of this qualitative study was to provide an in-depth understanding of mothers with young children’s perceptions and experiences that led to their initial engagement and sustained participation in group-based PA programs. Three themes were identified which focused on mothers with young children’s previous PA engagement, recruitment factors that engaged mothers with young children’s in group-based PA programs, and sustainable factors that maintained mothers with young children’s participation in group-based PA programs.

Overall, mothers with young children (in this study, children in the 0–5 age group) emerged as a significant opportunity for women to reconnect or initiate engagement in PA, regardless of prior experiences. Although underlying beliefs and perceptions play an important role in the decision-making processes of being physically active [20], there is also recognition that this is vastly influenced by social networks and experiences encountered during new phases of life (e.g. starting and raising a young family) [21]. For the mothers with young children in this study, it seems that social networks and new life phase experiences led to reconstructions of positions in life and previous ways of knowing. As a result, the women were able to change or reinforce their positive perceptions of PA during this life phase to make it a sustainable behaviour, which is contrary to other research on PA and mothers with young children which found that PA barriers outweighed enablers for mothers with children aged up to 12 months of age and that changes in PA behaviours in working mothers were not sustainable beyond 6 months [9, 22]. Perhaps, in this study, it was due to personal social support structures that led to recruitment into the group-based PA program and the social support structures within the group-based PA program that made the space welcoming for women and their child(ren), as well as providing a large number and a variety of sessions each day and week.

Each postpartum woman was subject to unique personal experiences relating to pregnancy(ies) and birth; however, the slow and progressive return to MVPA was similar for each woman in this study. This is interesting, as participants in other studies have described pressure to get back into shape quickly postpartum, often referencing specific influential people on social media, and therefore tend to increase the frequency and intensity of PA too quickly [23]. All participants in the current project mentioned the ‘6-week check-up’ as a critical moment for getting clearance for PA, but then depending on their birth experience, some of the women were able to access other health services to help support them to make appropriate decisions in relation to PA progression. This study supports previous research that has promoted the call for more interaction between hospitals, community health centres, women’s health physiotherapists, exercise providers, and policy makers to work together to educate mothers with young children and provide integrated services over an extended period [9, 24]. The fact that many women have relied on family and social connections to find their group-based PA program reinforces the need for postpartum health professionals and healthcare practitioners to utilise the embedded opportunities they have with service delivery to encourage regular participation in MVPA by having knowledge of and recommending local and suitable group-based PA programs or working with mothers’ to utilise family and social connections to PA programs that may already be in place [6, 9, 25]. Consistent with other studies, this study highlights the need for health professionals and healthcare practitioners to upskill in this area or connect with an integrated postpartum health model that will provide this information at an appropriate time for women [24, 26].

Interestingly, existing postpartum interventions have largely utilised flexible delivery methods that facilitate women’s engagement in PA on their own (e.g. SMS [27, 28], telephone [29], website [30], apps [31], home-based [32]). These were generally adopted following formative research expressing a preference for minimal face-to-face contact, no interest in or inability to join group-based PA programs, and to reduce costs of paying exercise providers [27]. However, this current research study suggests that face-to-face sessions, convenient locations, appropriate timing, group contexts, catering for babies and child(ren), reasonable ongoing costs, and a welcoming and supportive ethos make group-based PA programs a preferred option. This is consistent with more recent research suggesting that previously identified barriers to postpartum PA are either not relevant for all women or that there has been a recent societal shift in women’s focus after birth to consider the baby in everything they do [13]. Further, research has shown that doing MVPA with similar group members increases attraction and level of involvement with the group [33]. Future research focusing on mothers with young children needs to design group-based PA programs with face-to-face sessions that cater for babies and children and are offered at different times throughout the day and week.

To our knowledge, this is the first study to explore mothers with young children’s sustainable participation in group-based PA programs. Most previous studies have confirmed the positive effect of social support in enabling MVPA and the findings of this study builds upon that other work [9, 27]. However, in this study, the role of partner support was acknowledged but not as imperative for facilitating and sustaining PA participation mainly due to partners’ work commitments. Therefore, friends and associates, other family members, and the group-based PA program participants were the main form of social support for recruitment and continued engagement. Similar with Ellis et al. [33] work, participants in this study preferred to be active with groups of mothers because they were all in similar situations, could share experiences, gather advice and support relating to motherhood and children, and were more likely to create non-judgemental environments conducive for self-efficacy, mental health, wellbeing, and body image. These attributes align with self-determination theory (SDT) and show that when group-based PA programs satisfy three innate psychological needs of autonomy, competence, and relatedness, mothers with young children will be more likely to sustain their engagement in group-based PA programs [34].

Given the physical, social, and psychological benefits of engaging in regular PA [35], it is not surprising that the women in the study spoke of numerous gains, including positive thoughts and capabilities of the body, psychological stress relief, and social enjoyment. Importantly, the mothers with young children in this study were generally appreciative of their postpartum bodies, particularly its strength and functionality due to the body positive environment created within the group-based PA program. This finding supports recent research that stipulates the need for exercise providers to promote and implement body appreciation strategies to develop healthy body image among mothers with young children [10]. This ensures that mothers with young children sustain their engagement in PA and benefit socially and mentally rather than avoid PA or overexert themselves to alleviate appearance concerns.

The in-depth qualitative data collection methods and the larger number of participants recruited to the point data saturation was achieved (interview narratives providing the same information, defined as ‘informational redundancy’ [36]) in an area that is currently not well understood are a strength of this study. However, the generalisability of this study is limited due to the focus on the Australian context and an under-representation of less-educated, single, uniparous, and culturally diverse women. In addition, although this study recruited mothers with young children from several metropolitan and regional areas, this study does not account for rural variations in the provision and accessibility of group-based PA programs. Despite these limitations, this was the first study to focus on mothers with young children who regularly participate in group-based PA programs.

In conclusion, there are many factors that recruit and sustain mothers with young children’s engagement in group-based PA programs. Evidence from this study shows that mothers with young children have different perceptions and meanings of PA; however, they relish a welcoming and supportive environment that accommodates babies and young children, is affordable and convenient, focuses on building strength and functionality, and is non-judgmental. These types of exercise environments allowed mothers with young children in the present study to experience the social and psychological benefits of MVPA and helped them sustain their engagement. Future group-based PA and community sport programs, as well as workplaces with mothers as employees, should design PA interventions targeting these appropriate features, as well as connecting with local health professionals and practitioners to ensure that mothers with young children access group-based PA programs that are safe, designed to be developmentally appropriate, and are connected to women’s health specialists allowing for women to have direct access to help and support when needed.
